# The Importance and Perspective of Magnetic Resonance Imaging in the Evaluation of Endometriosis

**DOI:** 10.1155/2013/436589

**Published:** 2013-11-20

**Authors:** Agnieszka Bianek-Bodzak, Edyta Szurowska, Sambor Sawicki, Marcin Liro

**Affiliations:** ^1^Department of Radiology, Medical University of Gdansk, Debinki 7, 80-211 Gdansk, Poland; ^2^The Second Department of Radiology, Medical University of Gdansk, Smoluchowskiego 17, 80-214 Gdansk, Poland; ^3^Department of Gynaecology, Medical University of Gdansk, Kliniczna 1 A, 80-402 Gdansk, Poland

## Abstract

MR imaging is becoming increasingly important in the assessment of patients with endometriosis. Its multiplanar capabilities and superior soft tissue contrast are particularly useful in the detection of deep infiltrating endometriotic implants. Endometriosis, defined as the presence of endometrial glands and stroma outside the endometrium, is among the most common gynaecological disorders affecting women in their reproductive age. The diagnosis and evaluation of the extension of endometriosis are difficult only with physical examination and laparoscopy. According to the authors' personal experience, a special MRI technique and some imaging guidelines regarding different anatomical localizations of endometriosis are discussed. This review is a brief presentation of current evidence on the diagnostic accuracy of MRI in the evaluation of endometriosis concerning other diagnostic methods, the limitations of MRI and its essential usefulness for preoperative diagnosis of deep pelvic endometriosis, and future perspectives in monitoring this disease.

## 1. Introduction

Endometriosis is defined as the presence of endometrial glands and stroma outside the endometrium. The exact prevalence of endometriosis is unknown, but it is estimated that 10–15% of the female population in the reproductive age may be affected by endometriosis and that millions of women in the world suffer from this disease [1]. Over the last years, particular interest has been noted on the infiltrative form of the disease. Deep infiltrating endometriosis (DIE) is defined by the presence of endometrial implants, fibrosis, and muscular hyperplasia under peritoneum and involves vital structures such as the bowel, ureters, and bladder, as well as rectovaginal space. Deep infiltrating endometriosis is histologically defined as endometriotic lesion penetrating into retroperitoneal space or the wall of pelvic organs to the depth bigger than or equal to 5 mm, measured starting from the surface of the peritoneum [2].

The diagnosis and the evaluation of the extension of DIE are difficult with physical examination and laparoscopy. It requires palpation and the opening of the retroperitoneal space in order to confirm and to evaluate the extent of the lesions [2–5]. 

The correct preoperative diagnosis is fundamental in defining the best treatment strategy of endometriosis, especially in cases in which there are deep infiltration and bowel involvement, so noninvasive methods are required to obtain preoperative diagnosis of the location and extent of endometriotic lesions.

Two imaging modalities are used most often to identify and characterize lesions in endometriosis-transvaginal sonography (TVS) and magnetic resonance imaging (MRI).

Transvaginal ultrasonography (TVS) has been proposed as the first line-line imaging technique because it allows extensive exploration of the pelvis; it is well accepted and widely available.

However, the diagnostic value of transvaginal sonography (TVS) for the assessment of deep pelvic endometriosis and superficial peritoneal lesions is unclear, but it is surely recommended as the first imaging modality done to evaluate patients with suspected endometriomas and endometriosis of bladder [2, 6–8]. 

Rectal endoscopic sonography (RES) has been recommended for the detection of endometriosis in rectal, rectovaginal, vaginal, uterosacral or rectosigmoid locations [2, 6, 9–11]. 

Saba et al. proved that MRI and TVS show similar results in the identification of rectosigmoid endometriosis. They suggest that these methods may have complementary roles in the identification of rectosigmoid endometriosis depending on the site affected [8].

A completely new technique also taken into consideration for the diagnosis of DPE is saline contrast sonovaginography (SCSV) first described by Dessole et al. and then studied by Saccardi et al. In the diagnosis of rectal endometriosis, they found sensitivity of 66.7% for SCSV and MRI and specificity of 93.8% for SCSV and 95.8% for MRI [9].

Magnetic resonance imaging is now commonly used for the diagnosis of endometriomas and has a great advantage over other diagnostic methods because it allows a complete survey of both the anterior and posterior compartments of the pelvic at the same time. That is why extensive pelvic adhesions and ureteral involvement are both important indications for MR examination [2, 3, 6, 7].

Many papers have shown the usefulness of MRI in the diagnosis of deep endometriosis (Busard et al. 2010 [12], Saba et al. 2010 [6], Jarlot et al. 2008 [13], Chassang et al. 2010 [14], Loubeyre et al. 2009 [15], Onbas et al. 2007 [16], Bazot et al. 2004 [5], Maubon and Bazot 2007 [17], Roy et al. 2009 [3], Marcal et al. 2010 [4], Bazot et al. 2007 [11], Hottat et al. 2009 [18], Abrao et al. 2007 [19], and Saba et al. 2012 [8]).

In comparison to the most routinely performed MR study-at 1.5 T, pelvic MR imaging at 3.0 T provides the best results for the diagnosis and the preoperative staging of deep endometriosis [18]. In the study by Hottat et al., MR at 3.0 T enabled complete exploration of the pelvis with very high spatial resolution allowing especially the detection of thin structures such as uterosacral ligament, colon, and bladder walls. Summary of results of largest studies are presented in Table 1.

## 2. MRI Technique

It is recommended to perform MRI study in the first half of the menstrual cycle to increase the sensitivity of the detection of small hemorrhagic foci of endometriosis. 

The presence of hemorrhagic nodule is a very specific MR finding for endometriosis (with 100% specificity), but it is associated with low sensitivity because the nodules may be no longer hormone-related or the patient is in the third or fourth week of her cycle at the MRI examination and thus not bleeding-SABA. In order to decrease the angle of uterine anteversion, a moderately filled bladder is required, resulting in better evaluation of the pelvic structures. A half-filled bladder displaces the bowel superiorly leading to bowel movement artefacts reduction. Besides, an antiperistaltic drug such as glucagon (1 mg) should be administered intravenously or intramuscularly. To achieve better extension of vaginal and bowel walls, vaginal opacification (50 mL) and rectal opacification (150 mL) with ultrasound gel are recommended by some authors [15].

The mostly used standard imaging protocol included T2-weighted fast spin echo sequence performed in sagittal, coronal and axial planes and T1-weighted fast spin echo sequence performed in axial plane. The protocol also includes T1-weighted fast spin echo fat saturation sequence performed in axial and sagittal planes. For lesions smaller than 1 cm, T1-weighted sequences with fat saturation appear to be the most sensitive in detecting these lesions. A spectrally selective fat suppression sequence allows the differentiation between hemorrhagic or fatty content of cystic lesions (endometriomas or dermoid cysts) and the increased detection of small implants. 

Some authors avoid contrast-enhanced imaging due to the lack of a definite consensus concerning its indications. An accurate preoperative assessment of endometriosis extension, including deep implants and adhesions, has been demonstrated even without the use of gadolinium contrast medium [2]. Moreover, contrast enhancement, as seen in normal parametrium, small pelvic veins, and vascular or inflammatory peritoneal surface, could be wrongly interpreted as foci of endometriosis [15].

Recent technical advances in diffusion-weighted imaging (DWI) significantly enhanced the value of abdomen and pelvic MRI. The degree of restricted diffusion in biological tissues has been shown to inversely correlate with tissue cellularity and the integrity of cell membranes. DWI should be performed in axial plane using a single-shot echo planner imaging (EPI) sequence with SPIR technique and with *b* value of 500, 800, and 1000 [12].

### 2.1. General MRI Characteristics and Locations of Endometriosis

Endometriosis usually appears in three different forms: ovarian endometriosis (endometrioma), peritoneal endometriosis, and deep endometriosis.

The most common locations of endometriosis are the ovaries and the pelvic peritoneum. These different forms of presentation are inclined to have different imaging patterns that may cause specific imaging diagnostic problems [1, 2].

Several systems scores have been used to stage the extension of endometriosis also in relationship to different locations inside the pelvis. The most common system used to evaluate the disease is the revised classification system of the American Society of Reproductive Medicine (rASRM) which followed the American Fertility Society (AFS) score. Values are assigned to endometriotic lesions in the peritoneum and ovaries corresponding to the size of lesions and by analogy with adhesions on the ovaries and fallopian tubes. The summarized resulting point scores are classified into four grades of severity:

- Stage I (minimal) 1–5 points;
- Stage II (mild) 6–15 points;
- Stage III (moderate) 16–40 points;
- Stage IV (severe) >40 points.

It is relatively easy to use, but it does not take into account the involvement of retroperitoneal structures with deeply infiltrating endometriosis. For this reason, in 2005 the Enzian classification was developed as a supplement to the rASRM score in order to provide a morphologically descriptive classification of deeply infiltrating endometriosis. The Enzian classification currently has a poor level of international acceptance and is mainly used in German-speaking countries. 

The revised version combines morphological structures into compartments in order to simplify the system. Retroperitoneal structures are divided into the following three compartments:

- Compartment A, rectovaginal septum and vagina;
- Compartment B, sacrouterine ligament to pelvic wall;
- Compartment C, rectum and sigmoid colon.

Severity was rated in the same way for all the compartments as follows:

- Grade 1, invasion < 1 cm;
- Grade 2, invasion 1–3 cm;
- Grade 3, invasion > 3 cm [2].

**Figure 1:**
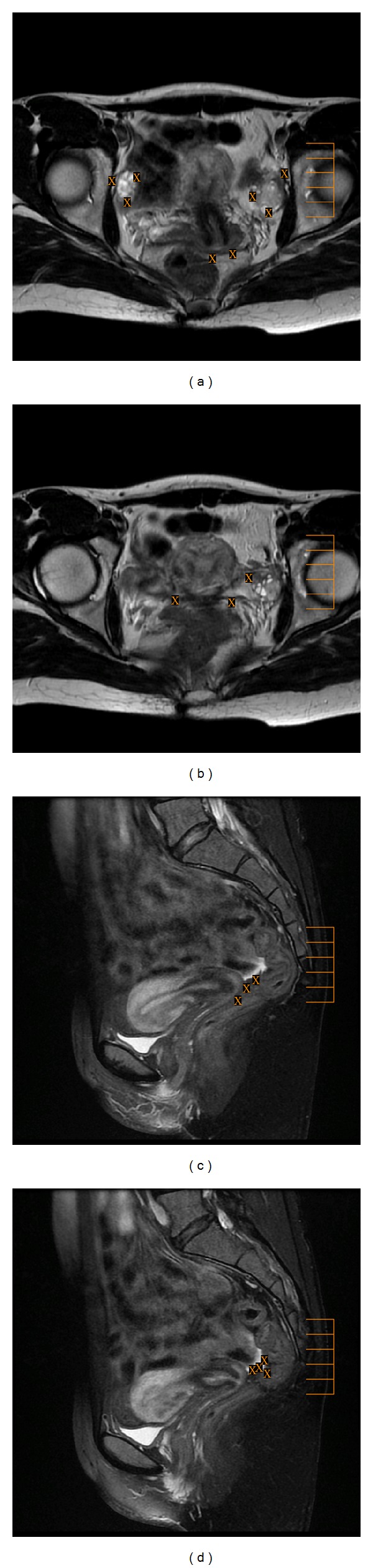
Axial ((a), (b)) T2-weighted fast SE images (repetition time msec/echo time msec = 4500/107) and sagittal ((c), (d)) T1-weighted fast SE images with fat suppression (TR/TE = 660/7.5) show the most frequent sites of involvement with peritoneal endometrial implants (X), such as the surface of the ovaries (a), uterus (a), pouch of Douglas ((a), (d)), uterosacral ligament (b), rectovaginal septum (c), and bowel (d).

The lesion locations are divided into parietal endometriosis of the abdomen, anterior subperitoneal endometriosis (bladder and vesicouterine pouch, ureters, round ligament, canal of nuck), and posterior endometriosis (uterosacral ligament, vaginal wall, posterior vaginal fornix, Douglas pouch and rectovaginal septum, rectal wall) (Figure 1). 

Parietal endometriosis of the abdomen is a rare location (0.03–2% of all cases of endometriosis). It can develop in different parietal locations, including the rectus abdominus, the umbilicus, the site of hysterectomy or caesarean scars, and the puncture sites of amniocentesis or trocar for laparoscopy [20–26]. 

The main risk factor for parietal endometriosis is prior abdominopelvic surgery although there were some cases of its occurrence in the absence of preceding trauma or surgery and metaplasia could be an explanation for its pathogenesis. The combination of ultrasound and MRI is useful for diagnostic workup, but correct preoperative assessment is achieved in about 20–50% of cases [20, 25]. 

Anterior subperitoneal endometriosis is divided into endometriosis of urinary tract, endometriosis of round ligament, and endometriosis of the canal of Nuck. Endometriosis can involve the urinary tract to up to 20% of cases and the bladder is most often involved [2, 7, 20]. 

The pathogenesis of bladder endometriosis is not well known: the reflux of menstrual flow through the fallopian tubes to the vesicouterine pouch might be an explanation. From there the endometrial cells could be implanted on the outer surface of the bladder and then increase in number and finally get to the mucosal surface of the bladder. MRI accurately diagnoses endometriosis of the bladder in 83–100% [5–7, 20] of the cases. 

**Figure 2:**
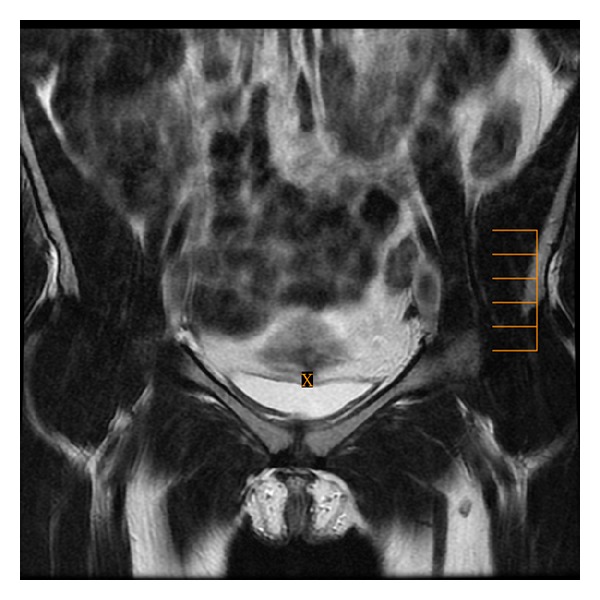
Coronal T2-weighted fast SE image (repetition time msec/echo time msec = 3020/101) shows the vesicouterine pouch, the potential site of anterior endometriosis.

The most common sites of lesions are the vesicouterine pouch and the bladder dome (Figure 2). 

MR imaging is characterized by localized or diffuse hypointense wall thickening on T1/T2 images. Certain authors recommend a systematic evaluation of urinary tract in patients with endometriosis because the prevalence of endometriotic lesions in urinary tract may be underestimated [2, 6, 7, 20, 27]. 

The prevalence of ureteral endometriosis ranges from 0.01% to 1% of all patients with the disease [20, 27]. Endometriosis of the ureter usually arises by extension from pelvic foci and ovarian endometriosis and may be due to ectopic implantation of endometrial cells along the lateral gonadal surface or ovarian fossa [20, 27]. 

 There are two types of endometriosis involvement of ureters:

- Extrinsic that represents 75–80% of the cases and is defined as the presence of endometrial tissue in the outer adventitia of the ureter that occurs as a nodule encasing the ureter;
- Intrinsic that represents 20–25% of cases and is defined as the presence of endometrial tissue in the mucosal and/or muscular layer of the ureter. The lesions are unilateral in 80% of cases and bilateral in 15–20% of cases, the majority of them present at the pelvic part of the ureter. MRI may show direct signs such as nodule or mass occurring in the ureter along its course or an indirect sign like ureteropelvic hydronephrosis superior to the suspected lesion [20].

Endometriosis of the round ligament is a rare finding with the prevalence of 0.3–0.6% of all women with endometriosis. In more than 90% of cases the lesion is on the right side. 

**Figure 3:**
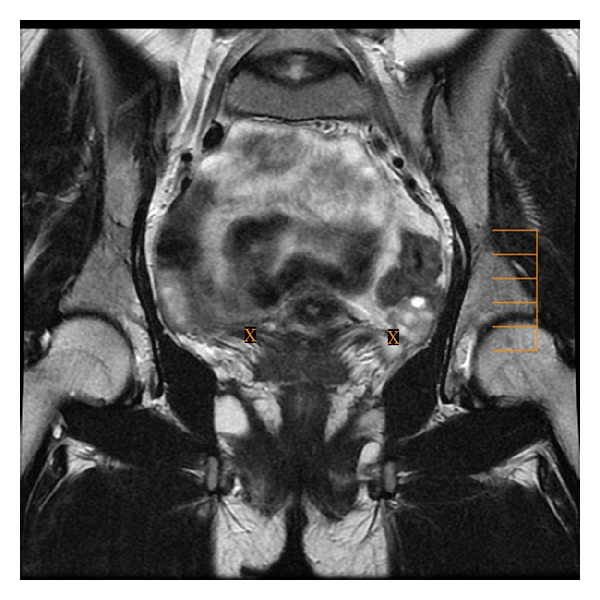
Coronal T2-weighted fast SE image (repetition time msec/echo time msec = 3020/101) presents both round ligaments, the potential site of anterior endometriosis.

The combination of ultrasound and MRI may be helpful in diagnosis, but in the majority of cases the diagnosis is established during the operation [20] (Figure 3).

The canal of Nuck is an embryological remnant of the peritoneovaginal canal near the labia majora in women and lies between the round ligament and the subcutaneous tissue. The endometriosis in this location is extremely rare (approximately 0.5% of cases). The mass is palpable in 96% of cases with predilection to the right side [20, 28–30]. Two patterns of Nuck canal endometriosis were described: type 1, predominantly cystic and type 2, predominantly solid with small-scattered cysts within lesion. All the cysts were hyperintense on T1-weighted images [31]. 

**Figure 4:**
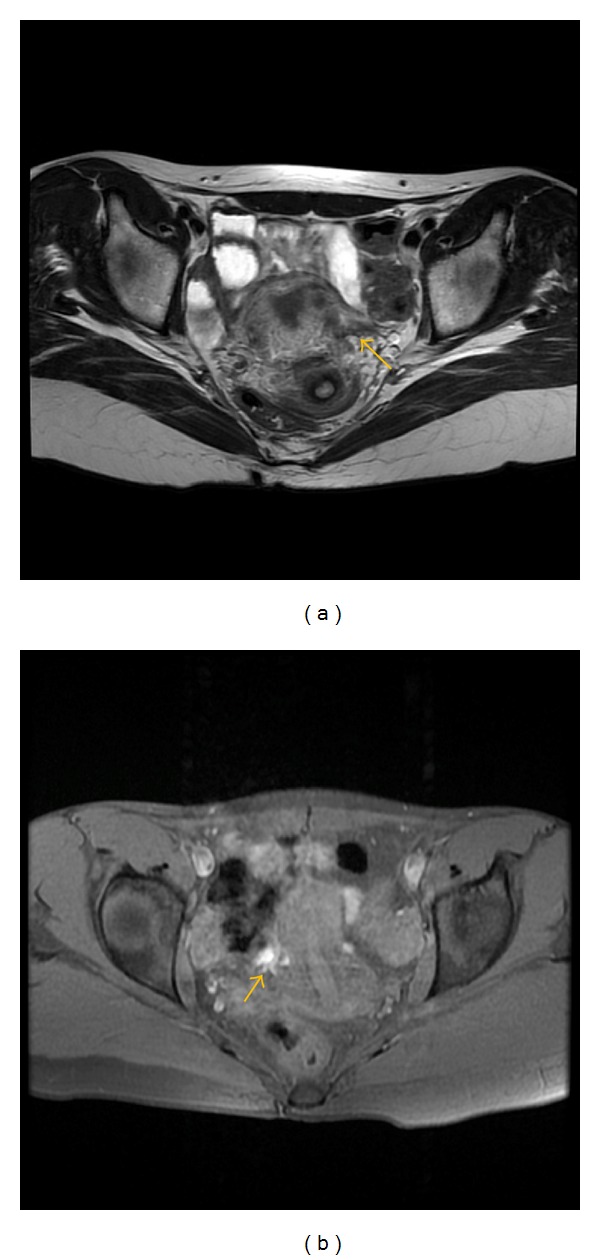
Axial (a) T2-weighted fast SE image (repetition time msec/echo time msec = 4640/102) shows the asymmetry of uterosacral ligaments with hypointense thickening and irregularity of left uterosacral ligament (arrow) and axial (b) T1-weighted fast SE image with fat suppression (TR/TE = 600/7.6) shows endometriotic hyperintense focus by right uterosacral ligament.

**Figure 5:**
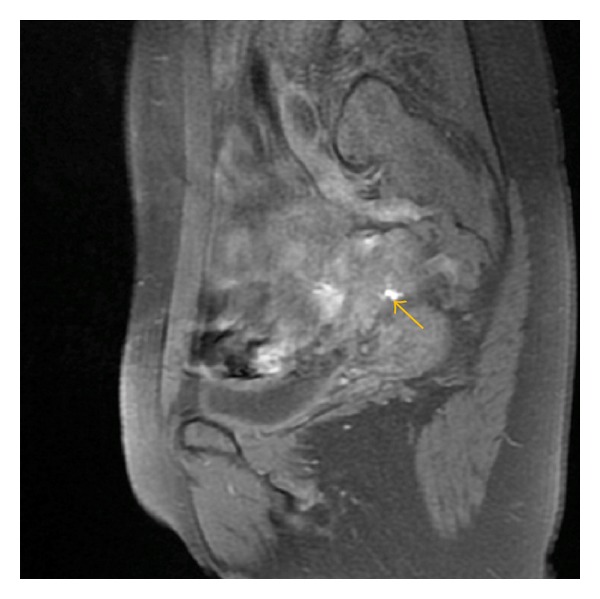
Sagittal T1-weighted fast SE image with fat suppression (TR/TE = 880/7.8) shows small hemorrhagic focus (arrow) in the rectovaginal septum (lesion type II).

Posterior endometriosis is concerned with uterosacral ligament, vaginal wall, Douglas pouch, and rectovaginal septum as well as rectal wall [1, 2, 32, 33]. Some studies revealed that the Douglas pouch, and uterosacral ligaments are the most common pelvic sites of endometriosis, and the frequency of endometriosis in the posterior Douglas pouch is equal to up to 56%, in uterine ligaments—69.2%, and in the vagina—14.5%. Uterosacral ligaments may be involved along their entire length. The most frequent is the involvement of the proximal medial portion of the uterosacral ligaments along the posterolateral margin of the cervix. Endometriosis of the uterosacral ligaments may directly extend to the rectum or to the lateral fornices [10, 34]. Some studies use the value of 9 mm for uterosacral ligament thickness to define involvement. Bazot et al. and Jarlot et al. used rather irregularity and asymmetry of uterosacral ligaments to define their involvement because they observed that ligaments measuring less than 9 mm may be involved (Figure 4) [5, 11, 13, 35]. Posterior cul-de-sac lesions include retroperitoneal lesions and intraperitoneal infiltrating lesions, which are divided into rectovaginal septum lesions (type I), posterior wall forniceal lesions (type II), and hourglass shaped lesions (type III). Lesions of type I are equal to 10% and occur between the posterior wall of the vaginal mucosa and the anterior wall of the rectal muscularis. Lesions of type II are equal to 65% and develop from the posterior fornix towards the rectovaginal septum (Figure 5). 

Lesions of type III are equal to 25% and occur when posterior forniceal lesions extended cranially to the anterior rectal wall. Obliteration of the pouch of Douglas is strongly suggested when retrocervical or retroisthmus nodules extend to the rectal wall.

**Figure 6:**
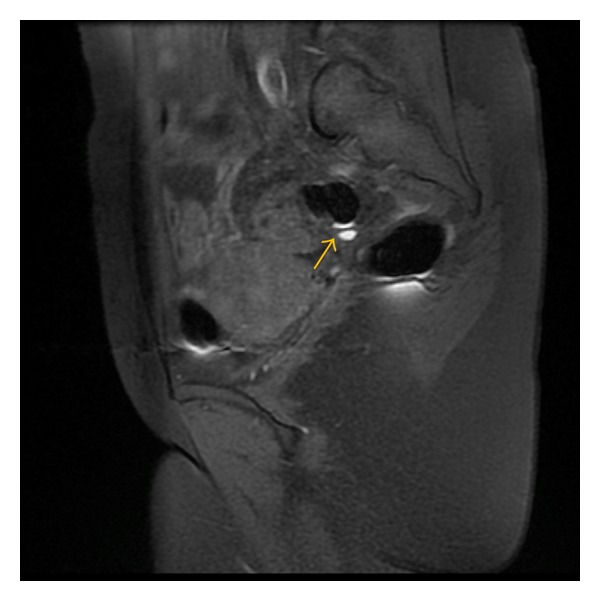
Sagittal T1-weighted fast SE image with fat suppression (TR/TE = 580/7.7) shows small hemorrhagic focus (arrow) in the rectal wall.

Solid endometriosis can involve the alimentary tract to up to 9.9% of cases. Rectosigmoid is the most common segment of the bowel involved (Figure 6) [15, 18, 36–38]. 

**Figure 7:**
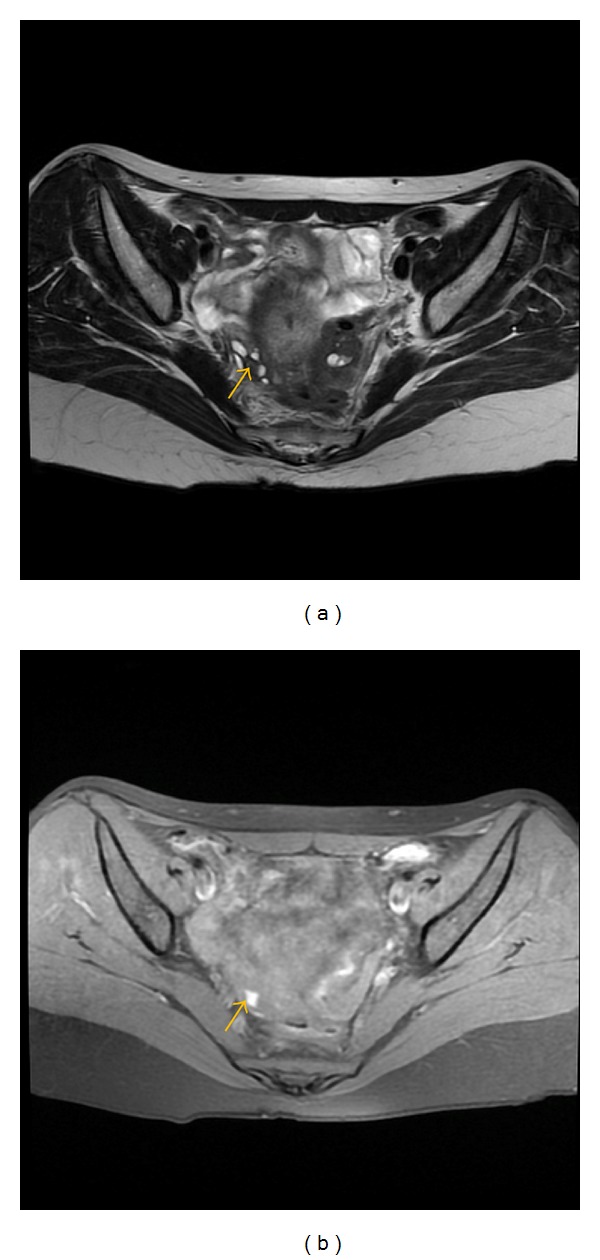
Axial (a) T2-weighted fast SE image (repetition time msec/echo time msec = 4640/102) shows typical hemorrhagic focus (arrow) as low signal intensity small lesion in the right ovary on T2-weighted image with corresponding high signal intensity on T1-weighted fast SE image (b) with fat suppresion (TR/TE = 600/7.6).

**Figure 8:**
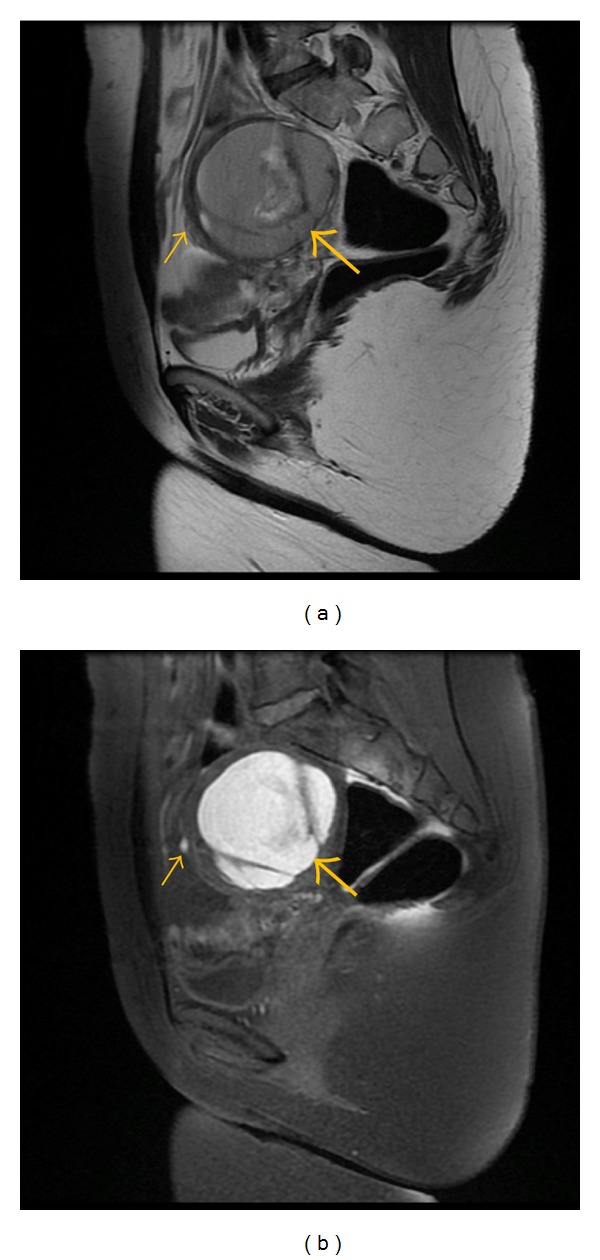
Sagittal (a) T2-weighted fast SE image (repetition time msec/echo time msec = 2940/66) and T1-weighted sagittal fast SE image (b) with fat suppresion (TR/TE = 580/7.7) show typical large endometrioma of the left ovary (large arrow) with satellite hemorrhagic focus in the anterior wall of this lesion (small arrow).

Classically endometriotic lesions in MR study represent spots of high signal intensity on T2-weighted images corresponding to endometriotic glands (exactly as the endometrium) or spots of high signal intensity on T1-weighted images corresponding to hemorrhagic foci in fibromuscular lesions. Cyclic bleeding of these lesions explains the T1 signal abnormalities (Figures 7 and 8).

Endometriotic lesions usually enhance after gadolinium contrast injection, but contrast-enhanced imaging cannot differentiate infiltrating lesions from other normal fibromuscular pelvic anatomic structures [16]. 

Deep infiltrating endometriosis is usually present as areas or nodules of low signal intensity on T2-weighted images with regular, irregular, indistinct, or stellate margins. This signal phenomenon might be explained by histologic findings. Endometriotic lesions consist mostly of smooth muscle cell proliferation and fibrosis that is the fibromuscular structure surrounding sparse ectopic endometrial glands. In several cases, little or no ectopic endometrial tissue is found. The involvement of anatomical structures such as the uterosacral ligaments or vaginal or rectal wall is suspected when these structures have a thickened or nodular appearance. [2–4, 13, 16, 18].

Recent technical advances in diffusion-weighted imaging (DWI) improved the value of body MRI and made it possible to calculate apparent diffusion coefficient (ADC) values as a representation of the degree of water molecular diffusion as well as perfusion within the assessed area. The degree of restricted water diffusion in tissues has been shown to inversely correlate with tissue cellularity and the integrity of tissue membrane. Many authors have reported decreased ADC values for many different malignant tumors.

The study by Busard et al. analysed the value of ADC for differentiating endometriosis infiltrating the bowel from colorectal carcinoma. Mean ADC value in DIE infiltrating the bowel was 0.8 ± 0.06 × 10^−3 ^mm^2^/s (range: 0.65–0.89 × 10^−3 ^mm^2^/s) and was significantly lower compared to mean ADC value in colorectal carcinoma which was 0.86 ± 0.06 × 10^−3 ^mm^2^/s (range: 0.74–0.98 × 10^−3 ^mm^2^/s, *P* = 0.02) [39].

DIE lesions infiltrating the bowel show hypointense signal intensity on high *b*-value DWI with corresponding low ADC values. In their study, Busard et al. found that ADC values in endometrial cysts showed considerable variation. The mean ADC value of endometrial cysts was 1.1 × 10^−3 ^mm^2^/s and was lower compared to the mean ADC value of functional ovarian cysts 2.14 × 10^−3 ^mm^2^/s. Their explanation was that ADC is almost linearly dependent on blood concentration and almost independent of the methemoglobin-related paramagnetic effect. Based on the published papers, it seems that ADC measurements can be helpful in the differentiation between the foci of endometriosis and other pathologies, but it is still necessary to prove their usefulness in daily practice. 

### 2.2. Limitations of MRI

Some of the factors limiting MRI performance for the detection of deep pelvic endometriosis are known. 

The sensitivity of MR technique may be reduced by bowel peristalsis, especially in cases of intestinal DIE, even after an adequate bowel preparation. 

Besides, there are three main anatomical reasons:

- retroflexed uterus;
- anatomical structure of the rectovaginal septum;
- vaginal and rectal walls that present MRI signal characteristics close to fibrous lesion.

The visualisation of the endometriotic involvement of the uterosacral ligaments can be limited in cases of retroflexed uterus. 

The vaginal walls normally collapsed and therefore difficult to evaluate. There are some difficulties in adequate visualisation of fibro-fatty components of rectovaginal septum. Endometriotic lesions are present classically as nodules and/or infiltrating masses, creating a continuum between different anatomical structures that distorts the pelvic anatomy making the normally distinct anatomical compartments indistinguishable [39]. Frequently these lesions are predominantly fibrous. In the study by Bazot, 100% of lesions were mostly fibrous and 61% were hemorrhagic. Imaging modality has to be able to detect fibrotic components that are hypointensive on T2-weighted MRI images and hypointensive on T1-weighted images [5, 11]. Therefore, briefly speaking, false-positive diagnosis may simply result from the misinterpretation of normal anatomic structures.

Pelvic MR imaging at 3.0 T could be the solution to the previously mentioned limitations of MR and provides the best results for the diagnosis and the preoperative staging of deep endometriosis [18]. In the study by Hottat et al., MR at 3.0 T made it possible to complete the exploration of pelvis with very high spatial resolution allowing especially the detection of thin structures such as uterosacral ligament, colon and bladder walls. Additionally, vaginal and rectal distension and opacification with ultrasound gel could help to delineate the cervix, vaginal fornices, and the anterior wall of the rectum and rectosigmoid junction [15] and MR imaging at 3.0 T should be the diagnostic method of choice in the evaluation of women with endometriosis.

## 3. Conclusion

MR imaging is a useful tool in the assessment of patients with endometriosis both as a stand-alone method and as a method complementary to transvaginal ultrasound. 

It enables precise mapping of deep infiltrating endometriotic implants. It could be acknowledged to be a reliable diagnostic tool especially at 3 T in preoperative evaluation of patients with deep pelvic endometriosis, being either a single diagnostic method or a method complementary to prior diagnostic procedures. 

Due to the lack of a definite consensus concerning contrast indications in patients with endometriosis, contrast-enhanced imaging is not necessary. An accurate preoperative assessment of endometriosis extension, including deep implants and adhesions, has been demonstrated even without the use of gadolinium contrast medium.

ADC measurements can be helpful in the differentiation between the foci of endometriosis and other pathologies, but it is still necessary to prove their usefulness in daily practice.

## Figures and Tables

**Table 1:** Overview of published studies using MRI for the evaluation of deep endometriosis.

Paper	Sensitivity	Specificity	PPV	NPV	*N* (number of patients)	Type of study	Imaging
(1) Bazot et al. 2004 [5]	90.3%	91%	92.1%	89%	195	Prospective	1.5 T
(2) Chamié et al. 2009 [40]	89.4%	92.3%	96.7%	77.4%	92	Prospective	1.5 T
(3) Roy et al. 2009 [3]	73%	93%	84%	88%	47	Retrospective	1.5 T
(4) Hottat et al. 2009 [18]	96.3%	100%	100%	97.6%	41	Prospective	3.0 T
(5) Jarlot et al. 2008 [13]	78%	70%	86%	58%	35	Prospective	1.5 T
(6) Saba et al. 2012 [8]	86%	73%	88%	24%	59	Prospective	1.5 T
